# The Potential of Children's Rearing Environment to Overcome Genetic Propensity for Low Reading Achievement

**DOI:** 10.1111/mbe.12332

**Published:** 2022-07-15

**Authors:** Leslie D. Leve, Gordon T. Harold, Jenae M. Neiderhiser, Misaki N. Natsuaki, Daniel S. Shaw, Jody M. Ganiban, David Reiss

**Affiliations:** ^1^ Prevention Science Institute, University of Oregon; ^2^ Faculty of Education University of Cambridge; ^3^ Department of Psychology The Pennsylvania State University; ^4^ Department of Psychology University of California, Riverside; ^5^ Department of Psychology University of Pittsburgh; ^6^ Department of Psychological and Brain Sciences George Washington University; ^7^ Child Study Center, Yale University

## Abstract

Genetic studies show that children's reading achievement is in part genetically influenced, and intervention studies show that reading achievement can be increased by environmental interventions. However, correlational and mean‐level analytic strategies are rarely integrated into achievement research, potentially leading to misinterpretation of results. The parent‐offspring adoption design offers a novel opportunity to examine the independent and joint roles of genetic and rearing environmental contributions. The sample included 344 adopted children in first grade and their biological and adoptive parents. Results indicated that adoptees' reading scores were correlated with their biological parents' scores, but not with their adoptive parents' scores, suggesting genetic influences. In addition, examination of mean scores indicated that adoptees' scores were significantly greater than their biological parents' (*p's* < .001) for all subtests, suggesting promotive effects of the rearing environment. This pattern was present even when biological parents scored >1 standard deviation below the biological parent mean on achievement.

## LAY ABSTRACT

We investigated reading achievement in first‐grade adopted children. Adoptees with higher reading scores had biological parents with higher reading achievement, but this association between parent and child was not present between adoptee and adoptive parents. However, adoptees' reading scores were more similar in mean value to their adoptive parents' scores than to their biological parents'. The findings underscore that childhood reading is genetically influenced, while also showing that one's rearing environment has an important effect that transcends genetic predispositions.

Understanding the joint roles of genetic and environmental factors underlying reading achievement in childhood is a significant issue for science and social policy that has long been a focus of psychological and educational research. There is unambiguous evidence from twin studies that reading achievement has a genetic component (see de Zeeuw, de Geus, & Boomsma, 2015 for a meta‐analysis). Molecular genetic studies also provide evidence of genetic influences on reading achievement, although findings regarding specific single nucleotide polymorphisms have not been consistently replicated (Carrion‐Castillo et al., 2016; Luciano et al., 2013). In general, polygenic scores (PGS) associated with overall educational attainment in independent general population samples have accounted for 7%–10% of the variance in specific cognitive performance (Lee et al., 2018). The findings from genetically informed studies have generated enthusiasm regarding education policy implications, with some researchers recently suggesting that children could be assigned to schools or academic subject areas partially based on their PGS (Plomin, 2019). However, because PGS show weak prediction, on their own they cannot inform education policy. Moreover, as behavioral genetic researchers have long emphasized (e.g., Plomin, 1999), the heritability of a cognitive trait does not mean that it is immutable. Rather, it is increasingly recognized that environmental contexts modulate the expression of heritability, and thus, genetic influences need to be investigated in tandem with potentially modifiable environmental factors (e.g., school, family). As a consequence, the field of reading development needs studies that simultaneously include both individual‐difference analyses that focus on correlations between genetically related and unrelated family members, and group difference approaches that focus on mean score differences between genetically related and unrelated family members (Turkheimer, 1991). Correlations index rank‐order similarities, which are different than mean‐level similarities.

To date, this dual analytic approach has not been applied to studies of achievement during the early elementary school years, when children have recently started formal schooling, and when reading achievement is malleable yet predictive of later reading and social‐behavioral outcomes (Ahmed, Tang, Waters, & Davis‐Kean, 2019; Fien et al., 2015; Vellutino, Scanlon, Zhang, & Schatschneider, 2008). A prospective adoption design with assessments of biological parents, adoptive parents, and the adoptee provides a unique opportunity to simultaneously examine both genetic and rearing environmental influences on reading achievement using a combination of individual differences and group differences approaches. Further, this design allows researchers to test whether the effects of genotype are uniform across rearing environments or not.

Examination of modifiable rearing environmental factors that promote reading success can be illustrated through evaluation of mean score changes in reading achievement. Mean scores are important to educators and academic policymakers alike because they are a cornerstone of curricular and educational decisions; individual and school‐level educational decisions are often made based on reading achievement test scores. Numerous school‐based interventions initiated in preschool and early elementary school have shown mean‐level improvements in children's reading achievement outcomes years later, suggesting the malleability of reading achievement in positive academic contexts (Mullender‐Wijnsma et al., 2016; Nix, Bierman, Domitrovich, & Gill, 2013; Reynolds & Temple, 1998; Stockard, Wood, Coughlin, & Rasplica Khoury, 2018). For example, an evaluation of the Chicago Child–Parent Center and Expansion Program with 426 children who participated in the program beginning in preschool found that program participation for 2 or 3 years after preschool and kindergarten was associated with significantly higher reading achievement up to seventh grade, after taking into account initial differences in achievement at kindergarten entry and at the end of kindergarten (Reynolds & Temple, 1998). It is clear from this study and from a host of meta‐analytic and review studies that reading interventions delivered during childhood demonstrate both short and long‐term positive effects on children's mean level reading achievement outcomes (e.g., Dietrichson, Bøg, Filges, & Klint Jørgensen, 2017; Galuschka et al., 2020; Graham et al., 2018; Suggate, 2016; Wanzek et al., 2018). One recent meta‐analysis of 25 intensive early reading interventions found a weighted mean effect size estimate of 0.39, suggesting that intensive early reading interventions result in positive outcomes for struggling readers in kindergarten through third grades (Wanzek et al., 2018).

Despite the impressive results from intervention studies, to our knowledge, reading intervention studies have not ascertained whether reading interventions work better for children who are genetically predisposed to be successful readers. Demonstration of malleability of children's reading achievement *in the context of genetic influences* on reading achievement would provide evidence for education policymakers of the benefits of providing quality education to all children, regardless of one's genetic propensities. Or, it might suggest that some children are impacted more by their rearing parents' reading achievement levels than are others, as a function of genetic influences. With either result, more specific information for education policymakers would be identified.

## THE PARENT‐OFFSPRING ADOPTION DESIGN

It is well known that reading achievement often runs in families, with inherited factors accounting for some but not most of this cross‐generational transmission (Johnson, McGue, & Iacono, 2006; Wadsworth, DeFries, Fulker, & Plomin, 1995). Another observation is that many familial and extra‐familial social risks and protective factors for reading achievement, such as family discord/coherence, parent–child hostility/warmth, and peer victimization (often experienced in school settings), are also shared within families (Harold, Leve, & Sellers, 2017). In biological families, associations between characteristics of the parent and characteristics of the child may result from underlying shared genetic characteristics that simultaneously influence both the trait in the parent and the trait in the child. When children are reared by genetically unrelated parents, this confound, known as passive gene–environment correlation, is removed (Plomin, DeFries, & Loehlin, 1977; Scarr & McCartney, 1983). For early educational interventions to be effective, they need to target social mechanisms that are genuinely promotive of positive outcomes. For example, a high level of parent involvement in promoting the child's reading skill development could be created or selected by parents, which could reflect genetically influenced features about the parent that also index high potential for reading attainment in the child because the parent and child are biologically related. For this reason, natural experimental research designs where the rearing environment is provided by adoptive parents who are genetically “independent” of their offspring can provide unique opportunities for identifying protective rearing and social environments that are independent of shared parent‐offspring genetic background. Such designs also provide an opportunity to examine the interaction between genetic influences on reading achievement and rearing parents' own reading achievement levels in predicting children's reading achievement.

To demonstrate how combining information from individual difference approaches (correlational approaches) with group difference approaches (mean score approaches) can advance the understanding of joint genetic and rearing influences on children's reading achievement, we used a parent‐offspring adoption design. This design also allowed us to test whether genetic influences had a consistent impact on children's reading scores, or whether the impact varied as a function of the adoptive parents' reading achievement levels. With a design that includes adoptees placed near the time of birth and the adoptees' biological and adoptive parents, we can examine associations between parent and offspring reading achievement that include shared genetic influences (biological parent‐adoptee associations) or that remove the effects of shared genes (adoptive parent‐adoptee associations). Specifically, our parent‐offspring design enables an examination of genetic influences on children's reading achievement—evidenced by significant correlations between reading scores of adoptees and their biological parents, relative to adoptive parents, while simultaneously examining their malleability—evidenced by adoptees' mean level reading scores having a smaller mean level difference with their adoptive parents, relative to the mean level difference with their biological parents.

## STUDY HYPOTHESES

Standardized assessments that assess multiple indicators of reading achievement were collected from adopted children, their biological parents, and their adoptive parents to test three study hypotheses. First, replicating prior genetically informed studies, we hypothesized significant associations between biological parent and adopted child measures of reading achievement, indicative of genetic influences. Second, we hypothesized that adopted children's mean scores on measures of reading achievement would be more similar to their adoptive parents' scores than to their biological parents', suggesting the importance of the early home and school environments for children's reading achievement. Third, we hypothesized that the similarity in mean reading scores between adoptees and adoptive parents would be present regardless of the child's genetic potential for reading achievement (i.e., regardless of whether the biological parents' mean reading score was below or above the biological parent mean for this sample). We further probed this hypothesis using a correlational approach by examining the interaction between biological parent and adoptive parent reading achievement on children's reading scores.

## METHOD

### Participants and Study Design

Participants were *N* = 344 linked sets of adopted children, adoptive parents, and biological parents participating in a longitudinal, multisite study of children adopted at birth in the United States, recruited in partnership with 45 adoption agencies from 15 states. The sample included *n* = 290 children in Cohort I of the Early Growth and Development Study (EGDS; Leve et al., 2019) and a subset of Cohort II children in EGDS (*n* = 54) who were invited to complete achievement assessments as part of a separate study (Early Parenting of Children [EPoCh]: Leve et al., 2018). A slight majority of the children in EGDS are male (57.2%), and the child race and ethnicity distribution are 54.5% non‐Hispanic White, 17.8% more than one race, 13.4% Hispanic or Latinx, 13.2% Black or African American, .5% American Indian or Alaskan Native, and <1% Asian, Native Hawaiian or Pacific Islander, or unknown.

In addition to the 344 adopted children, their biological mothers (*n* = 296), biological fathers (*n* = 96), adoptive mothers (*n* = 320), and adoptive fathers (*n* = 251) also completed the achievement assessment. The EGDS participants were recruited shortly after the birth of the adoptee and are currently being assessed longitudinally. Eligibility criteria for participation in the original EGDS study included: (1) the adoption placement was domestic, (2) the infant was adopted within 3 months of birth (*M* = 5.58 days, *SD* = 11.32 days), (3) the infant was placed with a nonrelative family, (4) both the biological parents and adoptive parents were able to read or understand English at an eighth‐grade education level, and (5) the infant did not have any major medical conditions. All assessments were administered in English and all children spoke English as a first language. This report focuses on an assessment of reading achievement conducted when children were in the first‐grade of elementary school and were approximately 7 years old.

The median total annual household income at the first‐grade assessment for adoptive families was between $100,001 and $125,000. The median educational attainment for adoptive mothers and adoptive fathers was at least a 4‐year college degree. Most adoptive mothers and adoptive fathers identified as non‐Hispanic White (91.3% and 90.6%, respectively); others were Black/African American (2.9% and 0.9%, respectively), Hispanic/Latinx (1.4% and 2.6%, respectively), more than one race/ethnicity (2.2% and 0.9%, respectively), and other/unknown (2.1% and 4.3%, respectively). Adoptive mothers and adoptive fathers were *M* = 44.01 (*SD* = 5.79) and *M* = 45.04 (*SD* = 5.77) years old, respectively, at the first‐grade assessment.

For biological families, the median total annual household income at the first‐grade assessment was $25,001–$40,000. The median educational attainment was a high school diploma for biological mothers and biological fathers. The majority of biological mothers and biological fathers identified as non‐Hispanic White (64.2% and 58.1.5%, respectively); others were Black/African American (20.9% and 25.7%, respectively), Hispanic/Latinx (9% and 4.1%, respectively), more than one race/ethnicity (3% and 10.8%, respectively), and other/unknown (2.9% and 1.4%, respectively). Biological mothers and biological fathers were *M* = 32.78 (*SD* = 4.97) and *M* = 35.51 (*SD* = 7.25) years old at this assessment, respectively. Further details about the study design and sample description have been described elsewhere (Leve et al., 2013, 2019).

An achievement assessment was conducted in the first grade (mean child age = 7.15 years old). Assessments were conducted in the family's home, by a trained assessor. All research activities were originally approved by the Institutional Review Boards of the Oregon Social Learning Center and the University of Oregon, and now fall under the Institutional Review Board of the University of Oregon. The study conforms to the recognized standards of the US Federal Policy for the Protection of Human Subjects. All adult participants provided consent, and all children provided assent prior to participating.

### Measures

#### Reading Achievement

Three reading‐related subtests from the Woodcock‐Johnson Tests of Achievement—III (WJ‐III; Woodcock, McGrew, & Mather, 2001) were individually administered to all child and adult participants. For biological parent and adoptive parent variables, where data were available for both parents, the mean of the two scores was used (*r*'s ranged from 0.16–0.34 for biological parent correlations and 0.21–0.34 for adoptive parent correlations). The WJ‐III test items are arranged in order of difficulty, and trained research assistants followed the standard administration protocols, which included the establishment of the floor (basal) for administration and continuation until a ceiling was reached. Standard scores were computed using the WJ‐III Compuscore and Profiles Program for each individual to classify individuals in relative standing. Standard scores take the individual's age into account, thereby allowing a standard comparison between individuals who vary in age (i.e., the adult and child participants in this study). Standard scores have a population‐normed mean of 100 and a standard deviation of 15. The three reading‐related subtests administered in the current study included: (1) Letter‐Word Identification, which measures the identification of letters (younger children) and words (older children and adults). The majority of items require an individual to read a list of words of increasing difficulty; (2) Word Attack, which measures an individual's ability to apply phonic decoding skills to pronounce unfamiliar words. The majority of items require that the participant pronounce nonsense works of increasing complexity; and (3) Reading Fluency, which measures the ability to read simple sentences quickly. It is a timed test with a 3‐min limit. Individuals are asked to read a series of sentences and indicate if they are true or false. Biological and adoptive parent data were only included if the adopted child completed the measure to enable more direct comparisons among the correlations by participant type.

#### Covariates: Openness of Adoption, Prenatal Obstetric Risks, Sex, and Parent Age

Analyses included two control variables specific to the adoption context that might bias the similarities between parents and the child (adoption openness and prenatal obstetric risks). Openness in the adoption, which reflects the degree to which adoptive and biological parents have knowledge of one another, was statistically controlled using a mean of adoptive mother and adoptive father ratings of the level of openness in the adoption at child age 7 (see Ge et al., 2008, for more information about this scale). Openness in the adoption has the potential to inflate similarities between biological parents and adoptees due to postnatal social interactions if contact between parties results in increased similarities between biological parent and child. In this sample, the full range of openness existed, ranging from very closed (no information) to very open (in‐person visits), although most families experienced at least some degree of openness. Prenatal obstetric risks (e.g., neonatal complications, prenatal drug use, prenatal exposure to toxins) were statistically controlled using a composite score that included five indices of risk derived from biological mother reports and medical record data pertaining to obstetric complications to create a weighted risk total score (Marceau et al., 2016). If not measured and controlled for, similarities between biological mother and adoptee may be due to prenatal environmental reasons rather than genetic. A wide range of prenatal obstetric risks existed in this sample, with most children experiencing some risks.

Additionally, we controlled for child sex (1 = male; 2 = female) given sex differences in reading achievement (Stoet & Geary, 2013). We also controlled for adoptive and biological parent age. Although the use of standard scores for parent and child achievement generally addresses age differences, it is nonetheless possible that parent–child similarities may be more pronounced among individuals who are more similar in age, based on cohort similarities in academic and educational practices that might exist at a population level.

### Analytic Approach

To test our first hypothesis, bivariate correlations were computed between the child's reading subtest score and the biological and adoptive parents' reading subtest scores, respectively. Correlations were adjusted for openness in adoption, prenatal obstetric risk, child sex, and parent age. Regression analyses then tested whether children's reading scores were predicted by biological parents' and adoptive parents' reading scores. To test our second hypothesis regarding mean level similarities, mean scores on reading subtests were computed for biological parent, adopted child, and adoptive parent, and an absolute value of the difference score between parent and child pairs was calculated.

To examine our third hypothesis, we categorized biological and adoptive parents into groups based on whether they had an achievement subtest score of 1 standard deviation above or below the mean for their group. We then examined the mean score for child and parent within these subgroups, and computed an absolute value of the difference score between parent and child within each grouping. As a follow‐up analysis using the correlational approach, we added an interaction term between biological and adoptive parent reading scores to the regression analyses from hypothesis 1, to see whether the effect of adoptive parent achievement varied as a function of biological parent achievement.

## RESULTS

### Hypothesis 1: Reading Score Correlations Between Child and Parent

Table 1 shows the correlations between adoptees and their biological and adoptive parents for the three reading subtests. Higher adoptee‐biological parent correlations relative to adoptee‐adoptive parent correlations denote genetic and/or prenatal effects; the opposite pattern denotes postnatal environmental effects. Table 1 confirms prior research that reading achievement is genetically influenced; the adoptee‐biological parent correlations were statistically significant for all three reading subtests, while none of the adoptee‐adoptive parent correlations were statistically significant. Fisher's *r*‐to‐*z* transformations indicated that the adoptee‐biological parent correlation was significantly different from the adoptee‐adoptive parent correlation for Letter‐Word Identification (*p* < .001) and Word Attack (*p* < .021). Using regression analysis, we tested whether children's reading scores were predicted by biological and adoptive parents' reading scores. In all three reading subtest models, biological parents' score was a significant predictor of the adoptees' score (*p* < .001*, p* < .001, and *p* = .005 for letter‐word identification, word attack, and reading fluency, respectively), but adoptive parents' score was not (*p* = .742, .706, and .132, respectively).

**Table 1:** Correlations Between Adopted Children and Their Parents' Reading Achievement Scores

	Correlation with adoptee	*p*	*n*
Letter‐word identification			
Biological parent	0.271^***^	<.0001	306
Adoptive parent	−0.003	.963	343
Word attack			
Biological parent	0.202^***^	<.0001	304
Adoptive parent	0.044	.424	341
Reading fluency			
Biological parent	0.157^**^	.007	304
Adoptive parent	0.070	.197	341

*Note*. Correlations partial out variance due to child sex, openness in the adoption, prenatal obstetric risk, and parent age.

### Hypothesis 2: Mean Level Reading Scores

Columns 1–3 in Table 2 present the means, standard deviations, skew, kurtosis, and *n* for biological parents, adoptees, and adoptive parents on the three reading subtests. There was no evidence of systematic skew or kurtosis. Paired t‐tests showed that for each reading subtest, adoptees' mean scores were significantly greater than their biological parents' scores (all *p*'s < .001). They were also significantly greater than their adoptive parents' subtest scores for letter‐word identification and word attack (*p's* < .001), but not for reading fluency (*p =* .833).

**Table 2:** Mean Scores on Reading Achievement Test Subtests for Biological Parents, Adoptee, and Adoptive Parents

	1	2	3	4	5	6	7
	Biological parent	Adoptee	Adoptive parent	Child/BP *M* if BP 1 *SD* below BP *M*	Child/AP *M* if AP 1 *SD* below AP *M*	Child/BP *M* if BP 1 *SD* above BP *M*	Child/AP *M* if AP 1 *SD* above AP *M*
Letter‐word identification (mean, *SD*, skew, kurtosis, *n*)	96.71	109.57	105.35	104.07/88.44 (Diffsc = 15.63) (*n* = 43)	108.33/99.43 (Diffsc = 8.90) (*n* = 63)	114.47/104.98 (Diffsc = 9.49) (*n* = 45)	110.08/111.27 (Diffsc = 1.19) (*n* = 48)
	8.27	13.92	5.92				
	−0.35	−0.25	−0.21				
	2.48	1.39	−0.85				
	*n* = 306	*n* = 344	*n* = 343				
Word attack (mean, SD, skew, kurtosis, *n*)	95.50	107.83	101.72	104.95/85.76 (Diffsc = 19.19) (*n* = 38)	105.72/94.62 (Diffsc = 11.10) (*n* = 54)	112.55/105.25 (Diffsc = 7.30) (*n* = 40)	107.64/108.82 (Diffsc = 1.18) (*n* = 55)
	9.75	11.25	7.10				
	−0.15	−1.29	0.19				
	0.94	3.35	−0.41				
	*n* = 306	*n* = 342	*n* = 343				
Reading fluency (mean, SD, skew, kurtosis, *n*)	98.74	105.68	105.46	100.39/88.14 (Diffsc = 12.25) (*n* = 54)	106.02/97.60 (Diffsc = 8.42) (*n* = 55)	108.64/109.33 (Diffsc = .69) (*n* = 53)	108.68/113.32 (Diffsc = 4.64) (*n* = 59)
	10.60	16.26	7.86				
	−0.22	−.44	.01				
	−0.51	−.43	.25				
	*n* = 306	*n* = 342	*n* = 343				

*Note*. AP = adoptive parents; BP = biological parents; Diffsc = absolute value of difference score.

### Hypothesis 3: Children's Mean Level Reading Scores When Parents Have Low or High Reading Achievement

Columns 4–7 in Table 2 show the adoptee and parent reading scores when parents were at least 1 standard deviation above or 1 standard deviation below the mean for that group of parents. In all cases, the adoptee's reading scores remained at or above the general population mean of 100, suggesting average to above‐average child reading performance. Absolute value difference scores between parent and adoptee are included for each parent–child comparison. In 9 of the 12 comparisons, the difference score for adoptive parent–child dyads was lower in magnitude than for biological parent–child dyads (paired *t*‐tests *p* < .001), suggesting that children' reading scores were generally more similar to their adoptive parents' scores, across both low and high levels of the adoptive parent and biological parent reading levels. The three exceptions were for reading fluency when biological parent and adoptive parent were at least 1 standard deviation above the mean, and for word attack and reading fluency when adoptive parents were at least 1 standard deviation below the mean and biological parents were at least 1 standard deviation above the mean. Follow‐up regression analyses indicated that the interaction between biological parents' and adoptive parents' reading scores was not significant when added to the models for hypothesis 1, suggesting that adoptive parents' reading achievement had a consistent promotive effect across different levels of genetic influences on children's reading scores.

## DISCUSSION

This study leveraged the unique features of the parent‐offspring adoption design to amplify prior work indicating that children's reading achievement is simultaneously genetically influenced *and* malleable. Although neither of these findings is surprising in its own right—behavioral and molecular genetic studies have shown that reading has a genetic component, and intervention studies have demonstrated the malleability of reading achievement—both of these aspects of children's reading achievement are typically not examined simultaneously. When only one aspect is the focal point, conclusions and policy recommendations can be misguided. More specifically, in biobehavioral approaches to medicine, genetic risk is typically a call to environmental action, whereas, in the field of education, genetic risk can be viewed as an impediment to learning. This paper integrates these approaches by jointly focusing on covariances and mean level differences to illustrate how environments can be an asset to reading achievement for all children, even in the context of genetic risk.

In brief, we replicated prior findings suggesting that children's reading achievement has a genetic component by showing that adoptees' reading scores were correlated with their biological parents' scores, even though they were not raised by them. As such, adoptees and their biological parents demonstrated *rank‐order* similarity in reading achievement. This replication is novel because in our adoption study the adoptees never resided with their biological parents, and therefore possible passive *r*GE effects as a source of biological parent–child reading achievement similarity were removed. We also showed that adoptees' mean‐level reading scores were more similar to their adoptive parents' scores than they were to their biological parents' scores, suggesting that the rearing environment can have a profound effect on children's reading achievement, even when biological parents have low reading levels. This rearing environment effect cannot be attributed to shared genetic influences (passive *r*GE), as adoptees were genetically unrelated to adoptive parents. Similar to other adoption studies (Capron & Duyme, 1989; Kendler, Ohlsson, Sundquist, & Sundquist, 2020; Scarr & Weinberg, 1983), the effect of the rearing environment in our study was advantageous. Building on Turkheimer's (1991) call to conduct analyses that eliminate the false dichotomy between genetic and environmental influences and instead estimate both influences, the current study unites approaches to show how children's reading achievement can be simultaneously genetic and malleable. Some of the unique analysis aspects include the age of the reading achievement assessments (first grade), the age of adoption placement (at or nearly at the time of birth), the focus on reading achievement and not on IQ or educational attainment, the inclusion of both biological and adoptive parents' reading scores, the removal of passive *r*GE from associations between the child's achievement score and both adoptive and biological parents' achievement scores, and the careful characterization of confounds that could potentially bias results.

The implications for educational policy and practice are significant because the findings suggest that even when a child might be at genetic risk for reduced reading achievement, rearing environments can have a positive effect on indicators of reading, resulting in higher reading performance than would be predicted by genetic factors alone. In this study, the mechanisms underlying this positive rearing environmental effect could be an advantageous home literacy environment, school selection and associated education curricula, and/or parental involvement in school—all of which are potential components of effective education policy warranting further study. Because children's achievement was positively impacted by their rearing environment regardless of genetic predispositions, there is no evidence to suggest that providing different educational programming for children as a function of their genetic makeup would be beneficial. Moreover, such practices have the potential to perpetuate inequalities in access to advantageous educational programs that may increase achievement levels. In other words, if such policies were implemented, children with higher genetic risk may not be given the same educational opportunities as those with lower genetic risk, even though they would benefit from exposure to an enriched educational environment at similar levels as other children.

Of note, this study assessed child achievement in first grade and heritability fluctuates across development (Plomin & Deary, 2015). Future assessments of this sample later in development will be able to examine whether similar patterns are present in adolescence. Further, adoptees' achievement scores could have been affected by unmeasured pre‐and perinatal factors. In addition, the current study did not include specific mechanisms within the rearing environment that might impact children's achievement, but extant research points to the home literacy environment and parental involvement in their child's schooling as important influences (Erdem & Kaya, 2020). Studies of children reared in bilingual homes and children with reading delays or reading disorders that are genetically influenced (e.g., dyslexia) are also needed to better understand the extent to which reading achievement scores are positively impacted by the environment in which children are reared. Taken together, the current results offer new insights into the importance of the early rearing environment for children's reading attainment as indexed by several indicators of reading skills, regardless of their genetic make‐up.
